# Giant lymphatic cyst of omentum: a case report

**DOI:** 10.1186/1757-1626-2-23

**Published:** 2009-01-07

**Authors:** Sanjeev Kumar, Nikhil Agrawal, Rahul Khanna, AK Khanna

**Affiliations:** 1 Department of General Surgery, Institute of Medical sciences, Banaras Hindu University, Varanasi, 221005, India

## Abstract

**Background:**

Omental cysts are rare abdominal lesions and are difficult to diagnose. Mostly they are detected incidentally during imaging studies performed for unrelated reasons.

**Case presentation:**

Presentation can be both acute and chronic. Acute presentations are usually due to complication in cyst. Imaging is helpful in excluding other causes of lump abdomen.

We encountered a case of giant lymphatic cyst presenting with abdominal swelling, clinically mimicking huge ovarian cyst.

**Conclusion:**

The goal of surgical therapy is complete excision of the cyst, and Omental cysts can be removed without endangering the adjacent bowel.

## Case presentation

**Figure 1:**
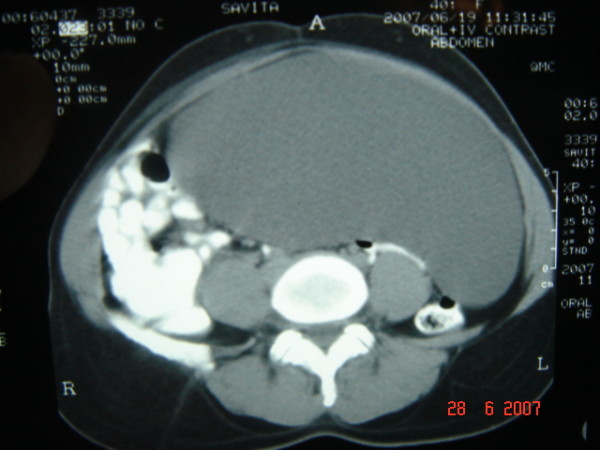
**CECT scan showing huge intra-peritoneal cyst**.

**Figure 2:**
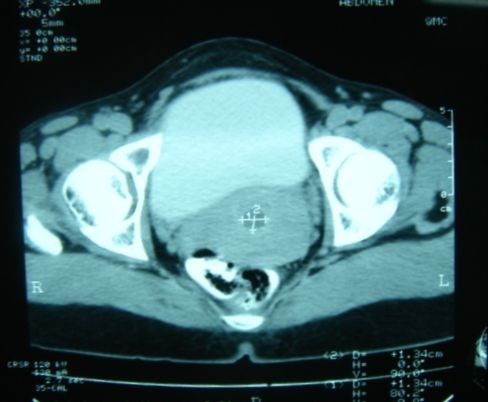
**CECT showing normal internal genitalia of the patient**.

A 42-year-old female presented to us with progressive distension of abdomen of one year duration. There was no history of anorexia or weight loss. It was associated with mild dull aching pain all over the abdomen. Her menstrual history was unremarkable. On examination, her abdomen was hugely distended. A vague soft, cystic lump was palpable involving whole of the abdomen. Routine blood tests were within normal limits. An abdominal Ultrasound examination was performed, and it showed a giant cystic lesion with internal echogenic particles and septations, extending from epigastrium to pelvis. All internal viscera were shifted postero-laterally. The origin of the lump could not be established. Provisional diagnosis of ovarian cyst was made and patient was advised to undergo CT scan. CECT showed the huge cystic lesion filling the whole peritoneal cavity with homogenous density (Figure 1). The internal genitalia were found to be normal (Figure 2). With these clinical and imaging findings, provisional diagnosis of omental or mesenteric cyst was made.

**Figure 3:**
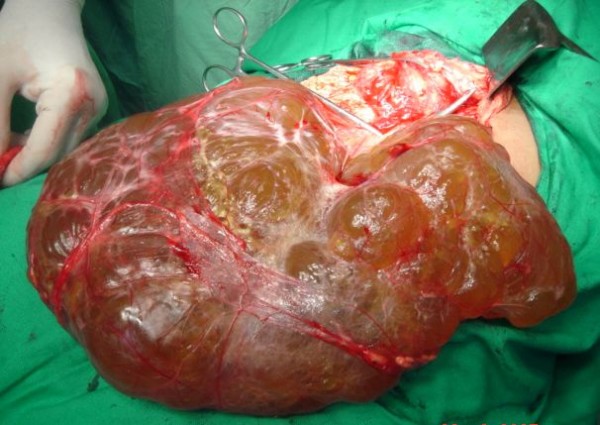
**Intraoperative photograph showing huge omental lymphatic cyst**.

Patient underwent laparotomy and a huge, soft cystic lesion arising from omentum was found. It was filling the abdominal cavity and has displaced all intraabdominal viscera posteriorly (Figure 3). It was excised. The gross pathologic appearance was of a huge thin walled cyst, with lymph filled cavities. Histopathological examination revealed that lesion was lined by single layered cuboidal cells and filled with lymph. These findings were consistent with lymphatic cyst of omentum.

## Discussion

Omental cysts are rare and are mostly derived from lymphatic tissue. They are present in the lesser or greater omentum and are lined by endothelium. Omental cyst occurs in all age groups, but most often presents in children and young adults [1]. Gairdner published the first report of an omental cyst in 1852 [2]. Omental cysts are thought to represent benign proliferations of ectopic lymphatics that lack communication with the normal lymphatic system. Other etiologic theories include (1) failure of the embryonic lymph channels to join the venous system, (2) failure of the leaves of the mesentery to fuse, (3) trauma, (4) neoplasm, and (5) degeneration of lymph nodes [3]. Omental cysts can be simple or multiple, unilocular or multilocular, and they may contain hemorrhagic, serous, chylous, or infected fluid. Omental cysts can be discovered as an incidental finding during laparotomy for another condition, or they can manifest as a chronic or acute abdomen. Chronic symptoms include progressive abdominal distension and pain. The mass may be huge, simulating ascites [4]. The most common mode of acute presentation is that of a small-bowel obstruction, which may be associated with intestinal volvulus [5] or infarction, hemorrhage into the cyst, infection, rupture, cystic torsion, and obstruction of the urinary and biliary tract. The imaging modality of choice is abdominal ultrasonography. Ultrasound demonstrates fluid-filled cystic structure, commonly with thin internal septations and sometimes with internal echoes from debris, hemorrhage, or infection. These can be confused with large ovarian cysts in females. Abdominal computed tomography (CT) scanning adds little additional information, although it can reveal that the cyst is not arising from another organ such as the kidney, pancreas, or ovary. The goal of surgical therapy is complete excision of the cyst [4]. Omental cysts can be removed without endangering the adjacent bowel. Prognosis is excellent.

## Consent

Written informed consent was obtained from the patient for publication of this case report and accompanying images. A copy of the written consent is available for review by the Editor-in-Chief of this journal.

## Competing interests

The authors declare that they have no competing interests.

## Authors' contributions

AKK and RK: operating surgeons. NA: collected clinical details including photograph, summarized the case history and prepared first draft. SK: conducted a literature search and prepared final draft. All authors read and approved the final manuscript.
